# Anticancer Effect of *Ferulago Mughlea* Peşmen (Apiaceae) on Cancer Cell Proliferation

**Published:** 2016

**Authors:** Bakar Filiz, Karakay Songül, Delimustafaoğlu Bostanlık, Fatma Gül, Kılıç Ceyda Sibel

**Affiliations:** a *Ankara University, Faculty of Pharmacy, Department of Biochemistry, 06100 Tandoğan, Ankara, Turkey. *; b *Ankara University, Faculty of Pharmacy, Department of Pharmaceutical Botany, 06100 Tandoğan, Ankara, Turkey.*

**Keywords:** *Ferulago mughlea*, Apiaceae, lyophilized extract, antiproliferative, cancer, prostate, colorectal carcinoma

## Abstract

*Ferulago* W. Koch. (Apiaceae) genus is represented by approximately 50 taxa throughout the world. *Ferulago* species are known as “Çakşır” or “Çağşır” in Turkey and mostly known for their aphrodisiac effects. However recent reports emphasize the activity of various *Ferulago* species against cancer, as well. The aim of this study was to investigate the effect of lyophilized extract of *F. mughlea *Peşmen, a species endemic for Turkey, on cancer cell proliferation. For this purpose human prostate (PC-3) and colorectal (SW-480) carcinoma cells were used to evaluate the antiproliferative effects of Ferulago W. Koch and the measurements were performed via MTT test. Lyophilized extracts obtained from aerial parts and the roots exhibited potent inhibitor effects on cell proliferation. Aerial part of the plant inhibited the proliferation of SW-480 cell at 48^th^ hour with a 0.119 mg/mL IC_50_ value.

## Introduction

Apiaceae is a very big and cosmopolite family throughout the world and it is understood that majority of the worldwide species diversity is concentrated in Asia (some genera are exclusively Asiatic) and among them the genus *Ferulago* W. Koch., which is represented by approximately 50 taxa throughout the world, is also found (1-3). The Asian countries with the greatest biodiversity are China, Turkey (Asiatic), Iran, Russia (Asiatic) and Kazakhstan (2). *Ferulago* species are known as “Çakşır” or “Çağşır” in Turkey (3) and according to the recent records, the genus is represented by 35 taxa in Turkey, 18 of which are endemics (4) and therefore Turkey is considered to be the gene center for the genus *Ferulago *(5). Though *Ferulago* species have traditionally been used in the treatment of hemorrhoids, intestinal worms, against ulcers, snake bites, sickness and headaches and also as sedative, stimulant and digestive; they are mostly known for their aphrodisiac properties (5-6). In the light of their traditional uses, many biological activity studies have been performed and various *Ferulago *species were found to possess cytotoxic (7-10), antimicrobial (11-14), antioxidant (15-18), immunomodulatory (19-20), antiangiogenic (21), anti-inflammatory (22) properties.

The results of biomedical research covering the last two decades years are quite in excess of information regarding the molecular events during carcinogenesis and potential signaling pathways involved in cancer progression. Recently many studies emphasizing anticancer, immunomodulatory, antiproliferative, antiapoptotic, cytotoxic activities of *Ferulago* species against different tumor lines (21-27) have been published, therefore we aimed to investigate the antiproliferative effect of lyophilized extracts of *F. mughlae* species on human prostate (PC-3) and colorectal (SW-480) carcinoma cells since cancer has become an important cause of morbidity and mortality in the world (28). 

## Experimental

*Plant material*

The plant material was collected from the below mentioned locality by one of the authors and identified by Prof. Dr. Hayri Duman (Gazi University, Faculty of Science, Department of Biology).The voucher specimen is kept in AEF (Herbarium of Ankara University Faculty of Pharmacy). 

Collection locality: C2: Marmaris National Park, Marmaris, Muğla, Turkey June 10, 2013 (AEF 26356). 

*Preparation of lyophilized extracts*

For the extraction procedure, 50,8207 g of roots and 104,7294 g of aerial parts were ground and macerated with 500 mL of distilled water each for 4 h at temperatures between 30-35 °C. Extracts of the roots and aerial parts were filtered and then lyophilized by using Christ Gamma 2-16 LSC Freeze Dryer and yielded 2,3319 g and 3,0816 g lyophilized extract, respectively. 

*Cell Culture*

Human prostate carcinoma cell line PC-3 (ECACC 90112714) and human colorectal carcinoma cell line SW-480 (ECACC 87092801) were purchased from Sigma Aldrich Co, Germany. PC-3 cells were cultured as 1:1 in Dulbecco’s modified Eagle’s medium (DMEM, PAA Laboratories GmbH, Coelbe, Germany) and Ham’s F12 (Lonza, Germany), whereas SW-480 cells were cultured with DMEM, with 10% fetal bovine serum (FBS, Lonza, Belgium), 1% antibiotics (penicillin/streptomycin, PAA, The Cell Culture Company, Austria) and 1% L glutamine (PAA, Austria) at humidified atmosphere (37 °C and 5% CO2).

*Assessment of Cell Proliferation*

The effect of lyophilized extracts on cell proliferation was determined by3-(4,5dimethylthiazol-2-yl)-2,5-diphenyltetrazolium bromide (MTT) assay by the method of Mossman (29) in modification of Kuzma *et al.* (30). A 180 μL volume of 5x104 cells was seeded and the cells were grown in a humidified atmosphere of 5% CO_2_ in air at 37 °C. Plant extracts were added in a concentration range between 10 mg/mL to 0.01 mg/mL final volume, and the plates were incubated for 24, 48 and 72 h. Control experiments were performed under the same conditions without addition of any extract. Following incubation, the culture medium was removed and exchanged for a fresh one. 20 μL of MTT solution (5mg/mL in PBS, Sigma) was added per well and incubated at 37 °C for 4 h. Metabolically active cells reduced MTT dye to formazan crystals. The blue MTT formazan was dissolved in DMSO (Merck). The extent of the reduction of MTT within the cells was quantified by measuring the absorbance at 540 nm on microplate reader (Thermo Scientific, MultiScan Go, Germany) and compared with untreated cells. The concentration of extracts required to reduce survival of cells by 50% (IC50) was determined from the graph of the amount of visible cells against test compound concentration.

*Statistics*

Statistical analyses were performed by SPSS (version 15; SPSS Inc). The data for in vitro analyses were expressed as % mean ± SD, and the analyses were performed within one-way Anova. A value of p< 0.05 was considered to be statistically significant.

**Figure 1:**
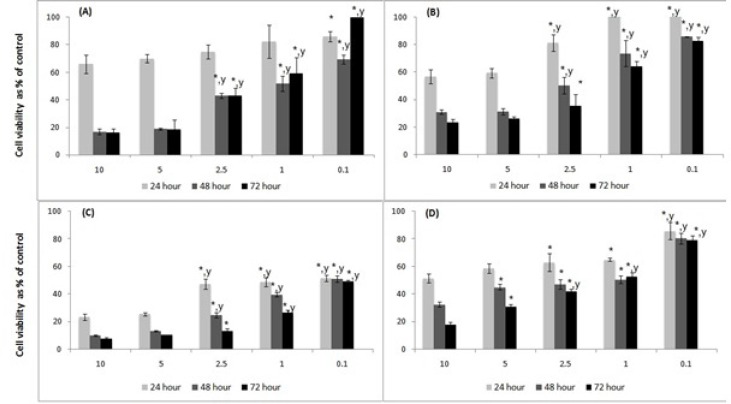
Cell viability determined by MTT Assay. Human PC-3 prostate carcinoma and human SW-480 colorectal carcinoma cells were seeded at 5x10^4^ cell/mL in complete DMEM:F12 (1:1) and DMEM medium, respectively, and treated with different concentrations of *Ferulago mughlae*, such as aerial parts (A), and roots (B) for PC-3 cell line and aerial parts (C), and roots (D) for SW-480 cell lines (C) for 24, 48 and 72 h. Data (mean ± SD) represent relative number of proliferating cells % in comparison to control (100%) of four independent experiments each performed in triplicate. The differences indicates ^*^ from 10 mg/mL and ^y^ from 5 mg/mL (p<0.05

## Results and Discussion

*Effects on cell proliferation*

In the study, the PC-3 human prostate carcinoma and SW-480 human colorectal carcinoma cells were treated with different concentrations of lyophilized *Ferulago* extracts and the cell viability were measured for 24, 48 and 72 h as described in the experimental part. The results of these measurements are shown in Figure 1.

Cell viability was significantly lower for all cell lines and lyophilized extracts at 10 and 5 mg/mL concentrations. The viable number of cells significantly decreased at 48^th^ and 72^nd^ hours. 

In the PC-3 cells, the aerial part of the plant exhibited the highest cytotoxic effect with 1.722 mg/mL IC50 value at 48^th^ h, and we observed significant inhibition of cell proliferation at 10 and 5 mg/mL doses (16.91±2.23, 18.80±0.61%, respectively, p<0.05). In the SW-480 cell line, the aerial part inhibited cell proliferation more effectively, similar with PC-3. The IC_50_ value was 0.119 mg/mL for the 48^th^ h, whereas it was out of count at the 72^nd^ h. 

Our results show that, although both parts had significant effects on inhibition of cell proliferation, the aerial parts of the plant had more potent effect compared to the roots. When we review the literature on the cytotoxic and anticancer effects of various *Ferulago *species, we can see that extracts obtained from different parts of *Ferulago* species (flower, leaves and roots) possess promising biological activities (8, 10, 19, 21, 26, 27). Cytotoxic properties of some coumarins such as isoimperatorin, xanthotoxin (10) and felamedin (7) were also demonstrated against various tumor cells lines. These coumarins are known to be present in many *Ferulago *species, therefore they may be present in the species that we have studied, and in a previous study we have isolated aforementioned compounds from *F. isaurica, *as well (31). As a result, we can conclude that this species has promising effects against the proliferation of cancer cells, and may represent a herbal alternative to synthetic drugs due to its coumarin content. Further studies are necessary to elucidate the mechanisms underlying these effects and also to determine the responsible constituent(s). 
